# Schistosome immunomodulators

**DOI:** 10.1371/journal.ppat.1010064

**Published:** 2021-12-30

**Authors:** Sreemoyee Acharya, Akram A. Da’dara, Patrick J. Skelly

**Affiliations:** Molecular Helminthology Laboratory, Department of Infectious Disease and Global Health, Cummings School of Veterinary Medicine, Tufts University, North Grafton, Massachusetts, United States of America; Joan and Sanford I Weill Medical College of Cornell University, UNITED STATES

## Abstract

Schistosomes are long lived, intravascular parasitic platyhelminths that infect >200 million people globally. The molecular mechanisms used by these blood flukes to dampen host immune responses are described in this review. Adult worms express a collection of host-interactive tegumental ectoenzymes that can cleave host signaling molecules such as the “alarmin” ATP (cleaved by SmATPDase1), the platelet activator ADP (SmATPDase1, SmNPP5), and can convert AMP into the anti-inflammatory mediator adenosine (SmAP). SmAP can additionally cleave the lipid immunomodulator sphingosine-1-phosphate and the proinflammatory anionic polymer, polyP. In addition, the worms release a barrage of proteins (e.g., SmCB1, SjHSP70, cyclophilin A) that can impinge on immune cell function. Parasite eggs also release their own immunoregulatory proteins (e.g., IPSE/α1, omega1, SmCKBP) as do invasive cercariae (e.g., Sm16, Sj16). Some schistosome glycans (e.g., LNFPIII, LNnT) and lipids (e.g., Lyso-PS, LPC), produced by several life stages, likewise affect immune cell responses. The parasites not only produce eicosanoids (e.g., PGE2, PGD2—that can be anti-inflammatory) but can also induce host cells to release these metabolites. Finally, the worms release extracellular vesicles (EVs) containing microRNAs, and these too have been shown to skew host cell metabolism. Thus, schistosomes employ an array of biomolecules—protein, lipid, glycan, nucleic acid, and more, to bend host biochemistry to their liking. Many of the listed molecules have been individually shown capable of inducing aspects of the polarized Th2 response seen following infection (with the generation of regulatory T cells (Tregs), regulatory B cells (Bregs) and anti-inflammatory, alternatively activated (M2) macrophages). Precisely how host cells integrate the impact of these myriad parasite products following natural infection is not known. Several of the schistosome immunomodulators described here are in development as novel therapeutics against autoimmune, inflammatory, and other, nonparasitic, diseases.

## Author summary

Schistosomes, or blood flukes, are long lived, parasitic worms that are found in the bloodstreams of >200 million people around the world. How the worms manage to survive in the face of attack by the mammalian host’s elaborate immune system is the subject of this review. The adult worms express a collection of enzymes on their surface that cleave important immune signaling molecules in the blood, and this could impede the ability of such molecules to awaken antiparasite immune cells. The worms also release a barrage of other proteins that directly interact with host cells to blunt their activation. The worms have nonprotein mediators too, including lipids, glycans, and nucleic acids (e.g., in the form of microRNAs) that can each affect immune cell responses. All of these metabolites may be used by the worms to bend host biochemistry to their liking and so ensure their survival. Several of the schistosome immunomodulators described in this work are in development as novel therapeutics against autoimmune, inflammatory, and other, nonparasitic, diseases.

## Introduction

Schistosomiasis (bilharzia) is an endemic and debilitating parasitic disease, caused by trematode worms called schistosomes (blood flukes) that currently afflicts over 200 million people in over 78 countries. The disease manifests primarily in 2 forms in humans—intestinal schistosomiasis, which can be caused by 2 main species, *Schistosoma mansoni* and *Schistosoma japonicum*, and urogenital schistosomiasis, caused by *Schistosoma haematobium* [1,2].

Adult schistosomes live in the vasculature of their mammalian hosts. Females can produce several hundred (*S*. *mansoni*, *S*. *haematobium*) or thousand (*S*. *japonicum*) eggs per day. Eggs that are released from infected hosts may pass into freshwater where they hatch to produce free-living, highly active larvae called miracidia. These penetrate intermediate freshwater snail hosts where they develop into sac-like creatures named sporocysts. These, in turn, give rise to many free-living, motile life stages called cercariae that exit the snail into the water where they seek a suitable mammalian host to infect [2–4]. During transdermal invasion of this final host, the parasites shed their swimming tails and transform into juvenile forms called schistosomula. These can stay in the dermis for up to 3 to 4 days before entering the circulatory system [5,6]. The young worms then travel through the bloodstream, first to the lungs, then the liver, and finally to the mesenteric veins (*S*. *mansoni*, *S*. *japonicum*) or perivesical veins (*S*. *haematobium*). Each schistosomulum matures and develops into either an adult male or a female worm. The adults mate and females begin laying eggs to complete the life cycle. Many parasite eggs leave the body through the host’s excretory system with feces or urine; however, numerous eggs remain permanently lodged within the liver and other tissues of the host [2]. The pathology of the disease, which can present as abdominal pain, bloody diarrhea, granuloma formation, hyperplasia, and fibrosis, is caused less by the presence of the worms in the bloodstream and more as a result of the immune response focused upon the accumulated eggs trapped in the tissues [2].

Schistosomes possess the unique ability of surviving within the body of an immunocompetent host for over 30 years [2,7]. How they do this is the subject of this review. Here, we discuss the role of a collection of molecules produced by the various life stages of the parasites that are either known, or are hypothesized, to modulate host immunity, negating its damaging effects and promoting worm survival. We focus on parasite molecules that are exposed at the parasite surface where they could interact directly with host biochemistry. Additionally, we examine the impact of parasite excretions/secretions (ES) on host immunity. We place less emphasis on the effect of soluble extracts/homogenates of whole parasites, since we consider that the impact of molecules that are naturally released by the worms is likely of greater physiological relevance compared to the impact of whole worm homogenates (that would not be seen in vivo).

The hallmark of the immune response toward schistosomes is the induction of a Th2 response in both humans and mice [2,4,8–11] and by the generation of regulatory T cells (Tregs) [12–15], regulatory B cells (Bregs) [16–18], anti-inflammatory, alternatively activated macrophages (AAMs, M2 macrophages) [19–21] as well as elevated IgE and eosinophil production [4,22]. Many of the effects of parasite immunomodulators, as reviewed here, act to drive these immunological outputs. By suppressing or altering the trajectory of the host’s immune response, released and host-exposed parasite molecules, as described in this report, are considered vital for parasite survival. Data from the mouse model of infection show that Th2 responses are also essential to allow the host to survive [8]. Immunoregulatory molecules are produced by all life stages of the worms and come in multiple forms including protein, carbohydrate, lipid, and nucleic acid.

### 1. Larvae in the skin: Cercariae and schistosomula

To help penetrate unbroken skin and establish infection, cercariae release an array of molecules from glands in their anterior called acetabular glands and head glands [23]. Such ES, collected in vitro within the first 3 hours of cercarial transformation, have been found to stimulate bone marrow–derived dendritic cells (BMDCs) to up-regulate expression of major histocompatibility complex (MHC) class II, CD40, and CD86 and to increase production of interleukin (IL) 12p40 and IL-6 [24]. Further, such DCs exhibit a potent capacity to drive Th2 polarization of CD4+ cells, as seen by increased IL-4 (but not interferon gamma (IFN-γ)) production [24]. While the Th2 immune response characteristic of schistosome infection predominates only after infection is established and egg laying begins, these data suggest that larval ES constituents could predispose DCs to promote Th2 responses over the Th1 phenotype before this time [11]. Note, however, that there is an early Th1 response in the mouse model prior to egg production [25]. This dichotomy highlights the difficulty in identifying a one-to-one correspondence between the impact of accumulated cercarial ES on isolated cells in culture (here, Th2 polarization) versus what is observed to happen in vivo in the early stages of a natural infection (a Th1 response).

Cercariae, but not schistosomula, are capable of inducing rat mast cell degranulation in vitro with the release of the vasodilator histamine [26]. This effect could assist the parasites following host penetration in vivo by facilitating entrance into the blood stream. While it is unclear how degranulation is incited, *S*. *mansoni* worms are known to express a 166 amino acid translationally controlled tumor protein (TCTP) homolog (**SmTCTP**) that, in recombinant form, can induce histamine release from a basophil/mast cell line [27]. The protein is widely expressed in all life stages within the vertebrate host and can be found in a schistosomula ES fraction [27] where it may help control blood flow.

*S*. *mansoni* skin-stage schistosomula ES can induce apoptosis of CD4+ and CD8+ lymphocytes (but not B cells) in a Fas-FasL-caspase8–mediated manner [28]. This activity is associated with a 23-kDa protein-designated *S*. *mansoni* apoptosis factor (**SmAF**) that has not been characterized [28]. In this manner, the parasites may drive T cell apoptosis to subdue initial damaging cellular responses in the skin. Additional uncharacterized and potentially immunomodulatory proteins have been identified in larval ES including venom allergen-like proteins (SmVALs) as well as proteases [23]. Indeed, an “elastase-like” serine protease in schistosomula ES (obtained after 3-day culture of transformed cercariae) was able to proteolytically cleave and inactivate IgE [29].

*1.1 Sm16, Sj16*. One component of *S*. *mansoni* cercarial ES that is thought to play a key role in the suppression of the host’s cutaneous inflammatory response is a nonglycosylated, 117 amino acid polypeptide designated Sm16 (also named SPO-1 or SmSLP) [23,30]. Sm16 is highly expressed in cercariae and in early transformed larvae; the protein is released from cercarial acetabular glands during host penetration and can additionally be detected in the schistosomulum tegument [31,32]. Sm16 is expressed in eggs and sporocysts too, but not in male or female adult worms [33]. The protein is a lipid bilayer binder that forms an approximately 9-subunit oligomer and attaches to the surface of diverse cell types in a polyanion-independent manner [31,32,34]. Based on sequence and gene organization considerations, Sm16 has been classified as a member of the helminth defense molecule (HDM) protein family, exclusively found in trematodes [33].

In recombinant form, Sm16 has been shown to induce production of anti-inflammatory IL-1 receptor antagonist a (IL-1ra) from human keratinocytes [35]. By competing with IL-1α and IL-1β for binding to IL-1 receptors, the released IL-1ra could potentially block IL-1–driven inflammatory responses in the skin. Sm16 also suppresses antigen-specific proliferative responses of lymphocytes isolated from the spleen and draining lymph nodes of the skin of *S*. *mansoni*–infected mice, and this is associated with inhibition of IL-2 secretion [35].

Recombinant Sm16 (rSm16) alone has no effect on T-lymphocyte activation, cell proliferation, or basal levels of cytokine production by whole human blood or monocytic cells [31]. However, rSm16 inhibits the cytokine response of T cells to the Toll-like receptor (TLR) 4 ligand, lipopolysaccharide (LPS), and the TLR3 ligand, poly(I:C) [31]. LPS-induced production of IL-6, tumor necrosis factor alpha (TNF-α), and IL-1β is potently suppressed by rSm16 [31]. While, as noted above, Sm16 induces IL-1ra production by keratinocytes, conversely, rSm16 inhibits the production of IL-1ra in LPS- or poly(I:C)-stimulated human blood cells [31].

Exposing bone marrow–derived macrophages to rSm16 prevents TLR4- and TLR3-, but not TLR2-mediated activation [36]. Sm16 is rapidly taken up by cultured macrophages where it is retained in early endosomes [36]. In addition to its ability to inhibit innate immune cell responses to select TLR ligands, Sm16 also blocks IFN-γ activation of macrophages in vitro, both by preventing IL-12p40 production and limiting nitric oxide (NO) production, thereby restricting their ability to become classically activated [36]. Thus, Sm16 has the potential to impair both innate and adaptive immune responses in infected hosts.

Evaluation of the transcriptome of human macrophages treated in vitro with synthetic Sm16 (encompassing amino acids 34–117) reveals that the peptide exerts significant impact on gene expression when administered either alone or in the presence of LPS [33]. Pathways prominently influenced are those involving transcription factors peroxisome proliferator-activated receptor (PPAR) and liver X receptor/retinoid X receptor (LXR/RXR); these play key roles in macrophage metabolism and are considered central to inflammatory responses [33].

The Sm16 homolog in *S*. *japonicum*, Sj16, has similarly been shown to exert immunomodulatory effects: rSj16 blocks the recruitment of leukocytes to the murine peritoneal cavity and up-regulates IL-10 and IL-1ra transcripts while down-regulating IL-12p35, IL-1β, and MIP-2 transcripts in peritoneal cells [37]. In addition, rSj16 inhibits LPS-induced activation of murine macrophage (RAW264.7) cells; the protein blocks NO production, decreases the levels of proinflammatory cytokines such as IL-1β, IL-6, IL-12, IL-23, TNF-α, as well as prostaglandin (PG)E2, while increasing IL-10 levels in these cells [38]. rSj16 also stimulates IL-10 production and inhibits LPS-induced BMDC maturation in a dose-dependent manner [39]. An N-terminal nuclear localization signal (NLS1) directs translocation of rSj16 to the nucleus of BMDCs to induce IL-10 production and inhibit BMDC maturation [39]. Finally, the protein can induce IFN-γ and IL-10 secreting Treg populations both in vitro and in vivo [40].

Knocking down expression of Sm16 in schistosomula in vitro results in a small (approximately 5%) decrease in mean parasite size compared to controls but has no significant impact on parasite survival or egg production in vivo [32]. In addition, vaccination with a truncated rSm16 does not result in any significant reduction in either parasite burden or parasite egg production [32].

Sm16 and Sj16 have both shown promise therapeutically; Sm16 can suppress cutaneous inflammation following intradermal administration of plasmid DNA encoding the protein [41], while rSj16 reduced the severity of chemically induced arthritis in rats [42] and protected against inflammatory colitis in a mouse model [43].

In addition to Sm16/Sj16 and other immunomodulatory proteins, all schistosome life stages examined (including cercariae and schistosomula) can synthesize a series of powerful, fatty acid–derived signaling molecules called eicosanoids [44]. These include prostaglandins PGE1 and PGE2 and 5- and 15-hydroxyeicosatetraenoic acids (HETEs) [45]. The parasites can additionally induce host cells to generate eicosanoids. For instance, live *S*. *mansoni* cercariae can induce PGE2 (as well as IL-10) release from human or mouse keratinocytes [46]. This effect is associated with an ES fraction <30 kDa in molecular size [46]. Considerable levels of PGE2 can be detected in homogenates of murine skin for up to 4 days following cercarial invasion [46]. Such parasite produced (or induced) biochemicals likely contribute to the worm’s ability to control host immunity and to establish infection, as described in more detail in the “Eicosanoids” section below.

**Fig 1:**
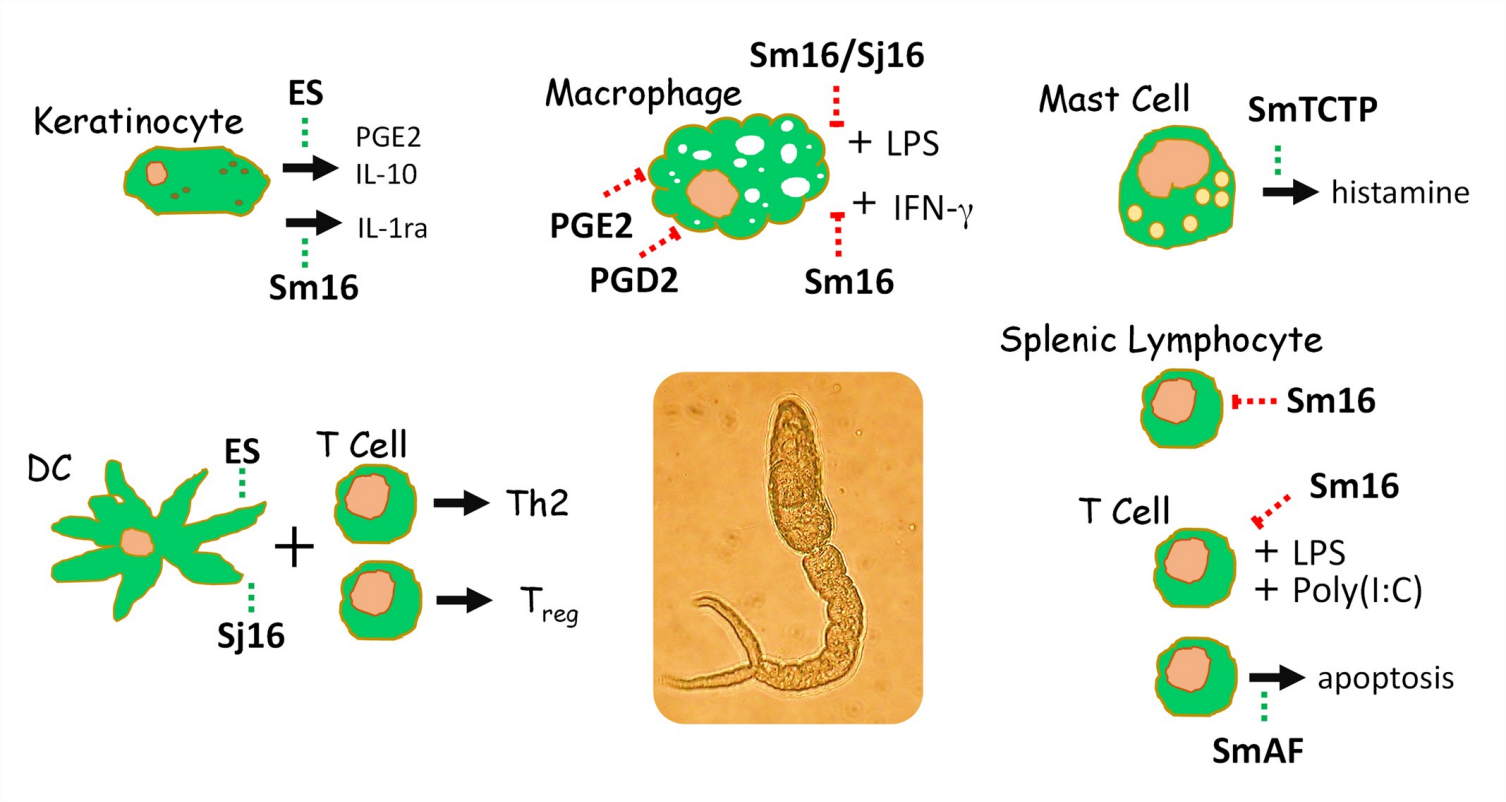
The impact of molecules (bold text) produced by schistosome larvae (cercariae and/or schistosomula) on the host cells indicated. An image of an *S*. *mansoni* cercaria is seen at the center. The green dashed lines indicate stimulatory effects, while red dashed lines indicate inhibitory effects. See text for details regarding the impact of individual schistosome molecules on specific cell types. DC, dendritic cell; ES, excretions/secretions; IFNγ, interferon gamma; IL-10, interleukin 10; IL-1ra, IL-1 receptor antagonist a; LPS, lipopolysaccharide; PGE2, prostaglandin E2; PGD2, prostaglandin D2 SmAF, *S*. *mansoni* apoptosis factor; SmTCTP, *S*. *mansoni* translationally controlled tumor protein.

Fig 1 summarizes the major reported impacts of cercariae/schistosomula ES components on a variety of cell types.

### 2. In the bloodstream: Schistosomula and adult worms

Most of the life of a successful schistosome is spent in the bloodstream of its mammalian host where it is potentially exposed to all components of host immunity, often for a very long time—the worms can survive in the human bloodstream for a decade or more [7]. An examination of adult worms in situ in experimental animals reveals that there is no evidence of overt inflammation around them—i.e., there is no accumulation of immune cells associated with the parasites in the vasculature [47]. How can these large foreign bodies in the blood stream avoid immunological scrutiny?

#### 2.1 Involvement of the tegument

One prominent aspect of the biological transformation that follows cercarial invasion of a vertebrate host is the shedding of the cercarial outer covering and the synthesis of a new host-interactive tegument that is bounded externally by a double-lipid bilayered membrane (a heptalaminate membrane). This surface is a major site for host–parasite interaction. Molecules found in the tegument play significant roles in immunomodulation and parasite survival. Next, we discuss the impact of some tegumental proteins on host immune cell activation and engagement.

*2*.*1*.*1 The roles of nucleotide metabolizing ectoenzymes (NMEEs)*—***SmATPDase1*, *SmNPP5*, *SmNACE***, *and* ***SmAP*. *SmATPDase1***. As schistosomes migrate through the mammalian bloodstream, it is hypothesized that they strain the vascular endothelium leading to the release of host stress signaling molecules collectively known as “alarmins” or “damage associated molecular patterns” (DAMPs) [48,49]. One example of a DAMP is adenosine triphosphate (ATP), which, in the extracellular environment, is a potent proinflammatory mediator. Extracellular ATP can act on multiple immunological effector cell types including neutrophils, macrophages, DCs, and lymphocytes (reviewed in [50–52]). Living worms can cleave exogenous ATP [48,53,54] via an ATP diphosphohydrolase that, in *S mansoni*, is designated SmATPDase1 [48]. This approximately 63-kDa protein possesses an N-terminal and a C-terminal transmembrane domain. It is detected in the adult tegument by immunolocalization [55]. It is identified in adult tegument extracts by proteomic analysis [56,57] and is available for surface biotinylation [58]. Recombinant SmATPDase1 has been shown to hydrolyze ATP in a cation-dependent manner and with a Km of 0.4 ± 0.02 mM [48]. It has been hypothesized that surface SmATPDase1, by cleaving extracellular ATP, mitigates its proinflammatory tendencies, and this promotes worm survival [59].

Living worms have additionally been shown to cleave exogenous ADP [48,53,54], and rSmATPDase1 has also been shown to cleave this metabolite with a Km of 0.25 ± 0.02 mM [24]. ADP is a potent activator of platelets, and these have been shown to be directly toxic to schistosomes (reviewed in [60]). For instance, platelets recovered from infected rats can kill schistosomula in vitro [61]. In addition, platelets from uninfected rats can kill larval schistosomes if they are first exposed to selected activators (e.g., serum from infected rats, C-reactive protein, TNF-α or TNF-β) [62,63]. Platelet antimicrobial effector molecules (e.g., platelet microbicidal proteins) may be responsible for damaging schistosomes [60]. SmATPDase1-directed cleavage of extracellular ADP may, therefore, block platelet activation and help schistosomes survive.

*SmNPP5* and *SmNACE*. A second schistosome nucleotide metabolizing ectoenzyme, designated SmNPP5, has also been reported to cleave exogenous ADP and, like SmATPDase1, could also assist in blocking ADP-driven platelet activation [64]. SmNPP-5 is an approximately 53-kDa polypeptide belonging to the phosphodiesterase/diphosphohydrolase protein family [65]. The protein possesses a single C-terminal transmembrane domain and is expressed exclusively in the intramammalian life stages [65,66]. SmNPP5 is expressed highly in the tegument of the intravascular life forms [66]. Proteomic studies confirm that the protein is found in tegument preparations and is available for surface biotinylation [56,58].

In addition to ADP, rSmNPP5 can cleave nicotinamide adenine dinucleotide (NAD) [67]. NAD-driven ribosylation of the host cell surface receptor P2X7R leads to cellular apoptosis, a phenomenon known as NAD-induced cell death (NICD) [68]. Since Tregs express high levels of P2X7R, they are especially prone to NICD [69]. SmNPP-5 has been shown to be able to prevent NAD-induced apoptosis of T cells in vitro [67]. This effect could promote Treg survival and so create a more immunologically hospitable environment for the worms in vivo. SmNPP5 is essential for schistosomes, since parasites whose SmNPP-5 gene is suppressed are severely impaired in their ability to establish infection in experimental animals [66]. In addition to SmNPP5, adult schistosomes express a second host interactive, NAD-catabolizing enzyme in their outer tegument. This approximately 35-kDa glycoprotein is designated SmNACE, and it is a member of the ADP-ribosyl cyclase family [70]. SmNACE may contribute to the ability of live worms to dampen levels of exogenous NAD and so further prevent NICD.

*SmAP*. Yet, another schistosome nucleotide–metabolizing ectoenzyme is alkaline phosphatase, SmAP, an approximately 62-kDa glycosylphosphatidylinositol (GPI)-anchored protein that is expressed in the tegument as well as in the internal tissues of the adult worms [71–73]. Tegumental proteomic analysis confirms that SmAP is found in the schistosome surface membranes [56,57] and is available for surface biotinylation [58]. The SmAP gene is most highly expressed in intravascular parasite life stages. Recombinant SmAP has been shown to dephosphorylate adenosine monophosphate (AMP) to generate adenosine [72]. Extracellular adenosine is a potent immunosuppressant; it has been shown to be capable of dampening many facets of a host’s inflammatory response [50,74–76]. For example, adenosine can inhibit proinflammatory cytokine production in macrophages and monocytes [77,78], attenuate the proliferative and cytotoxic responses of activated T cells [79,80], and inhibit neutrophil degranulation [81]. The ability of living schistosomes to cleave exogenous AMP and generate adenosine is largely abolished when SmAP gene expression is suppressed following RNAi treatment targeting the gene [72]. Such findings lend support to the hypothesis that schistosome surface SmAP could dampen host immune responses against the parasites by producing the immunosuppressant adenosine.

**Fig 2:**
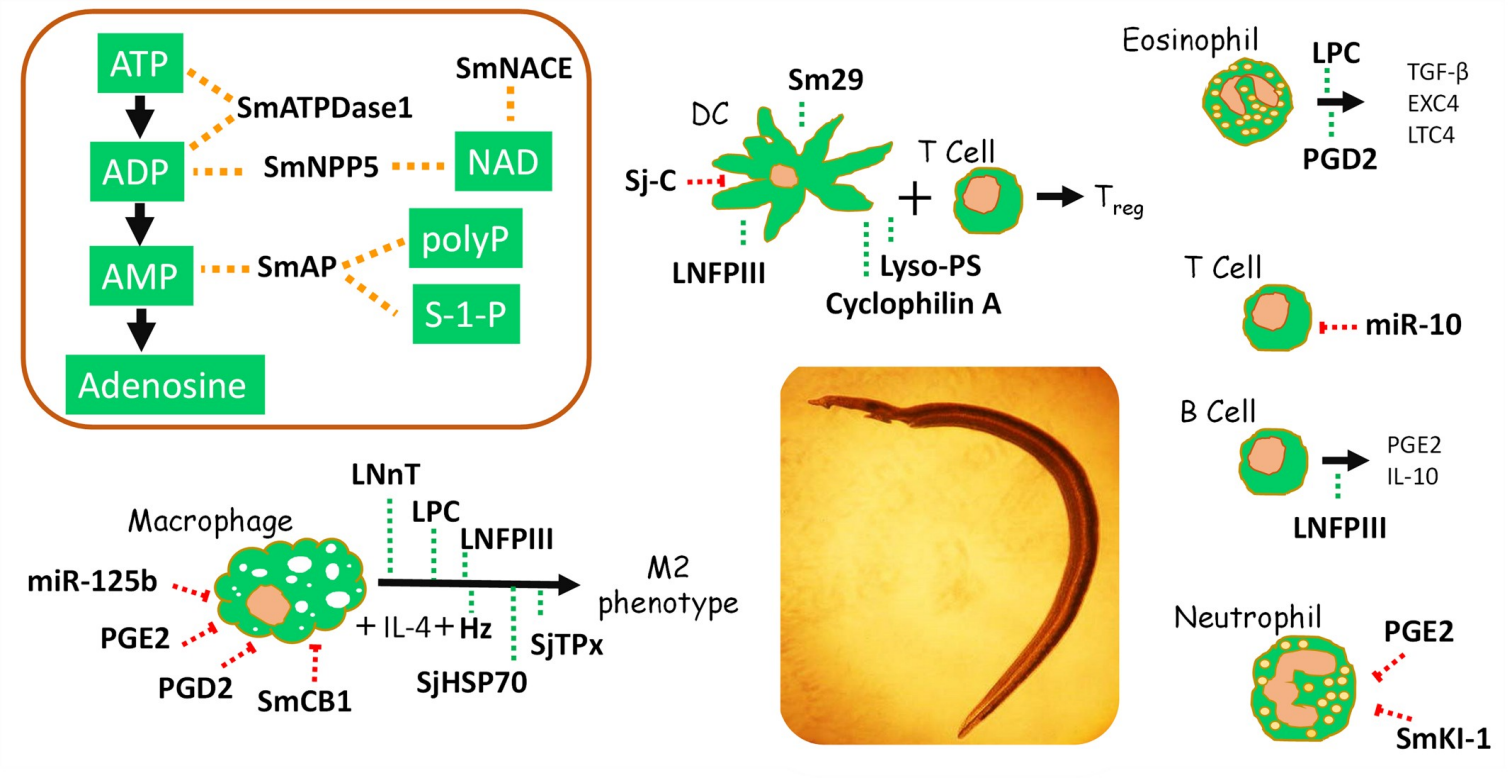
The impact of molecules (bold text) derived from intravascular stage schistosomes (schistosomula and adults) on host cells and metabolites, as indicated. The box (top left) lists *S*. *mansoni* tegumental NMEEs and, connected by dashed yellow lines, the host signaling molecules they have been shown to cleave (white text). The green dashed lines indicate stimulatory effects on immune cells, while red dashed lines indicate inhibitory effects. An image of an *S*. *mansoni* adult male is seen at center. See text for details regarding the impact of individual schistosome molecules on specific cell types. ATP, adenosine triphosphate; ADP, adenosine diphosphate; AMP, adenosine monophosphate; DC, dendritic cell; Hz, hemozoin; LNnT, lacto-N-neotetraose; LPC, lysophosphatidylcholine; NAD, nicotinamide adenine dinucleotide; NMEE, nucleotide metabolizing ectoenzyme; PGE2, prostaglandin E2; PGD2, prostaglandin D2.

As illustrated in Fig 2 (box, upper left), the worms possess the ability to efficiently convert any exogenous ATP into adenosine in the following manner: ATP can first be cleaved to ADP by SmATPDase1, ADP could be then converted to AMP by SmATPDase1 and/or SmNPP5, and, finally, AMP could be converted to adenosine by SmAP. Thus, the proinflammatory DAMP ATP may be expeditiously converted into anti-inflammatory adenosine by the intravascular worms via the concerted action of these 3 tegumental ectoenzymes.

Recombinant SmAP has been revealed to be capable of cleaving other important host signaling molecules such as sphingosine-1-phosphate (S1P) [82]. In the extracellular environment, S1P impacts vascular permeability and inflammation [83–86]. By degrading S1P, SmAP could help to limit the level of this bioactive lipid in the environment of the parasites and so restrict its ability to direct parasite-damaging immunity.

Another metabolite that intravascular schistosomes can hydrolyze is polyphosphate (polyP)—an anionic, linear polymer of inorganic phosphates that is produced and released by immune cells as well as by activated platelets and that induces proinflammatory pathways [87–89]. Recombinant SmAP can cleave polyP and with a Km of 6.9 ± 1 mM [87]. Parasites whose SmAP gene has been suppressed by RNAi are significantly impaired in their ability to hydrolyze polyP [87]. Therefore, SmAP-mediated cleavage of polyP, in addition to S1P and AMP, may contribute to the ability of schistosomes to generate an anti-inflammatory perimeter around them and so promote their survival in the otherwise hostile intravascular habitat.

Fig 2 (box, upper left) summarizes the potential impact of the 4 intravascular stage, *S*. *mansoni* tegumental NMEEs discussed above (SmATPDase1, SmNPP5, SmNACE, and SmAP) on selected host signaling molecules.

*2*.*1*.*2 Other tegumental proteins*. The tegument contains other proteins that are likely immunomodulatory. For example, *S*. *mansoni* tegumental proteomic analysis reveals the presence of 2 proteases belonging to the calpain superfamily (**SmCalp1** and **SmCalp2**) at the host-interactive surface [56,58], and a 28-kDa membrane-bound serine protease on schistosomula has been reported [90]. Following schistosome incubation in serum, cleavage of selected host proteins is detected, including components of the complement cascade [91], and it is possible that tegumental proteases are responsible [92,93].

Other schistosome proteins that have been proposed to play roles in protecting the worms from complement-mediated attack include a C2 binding protein (**SmTOR**), a C3 binding protein (unidentified) and a C8/C9 binding protein (**paramyosin**) [94–98]. However, since none of these proteins has been identified in multiple analyses of the worm’s tegumental proteome [56–58], how these proteins might exert an anticomplement protective effect in vivo is unclear.

Paramyosin has additionally been reported to be an immunoglobulin-binding protein [99]. In addition, several other proteins, some located in the tegument of *S*. *japonicum*, were also found to bind to immunoglobulin Fc domains, and it has been suggested that this blocks attachment of other host immune effectors [100].

Intravascular schistosomes express in their tegument homologs of the host GPI-linked membrane glycoprotein CD59—a key inhibitor of the complement membrane attack complex [101]. In *S*. *mansoni*, these are **SmCD59.1** and **SmCD59.2** [102]. Functional studies performed with these homologs provide no evidence for complement inhibiting activity and their role at the host/schistosome interface remains unclear [102].

One final capability of the schistosome surface of relevance here is that it can recruit host complement regulatory proteins such as delay-accelerating factor (**DAF**) [103,104]. It has been reported that DAF, a glycoprotein that can dissociate multiunit C3 convertases into their component parts to inactivate them, can be detected on the surface membranes of schistosomes [103,104]. The mechanism for transfer of DAF to the parasites is unknown.

*Sm29*. **Sm29** is a membrane-bound 191 amino acid glycoprotein found on the tegument of lung-stage and male and female *S*. *mansoni* adult worms [105]. Recombinant Sm29 is reported to induce the maturation and activation of human monocyte-derived DCs (MoDCs), as seen by increased expression of the cell maturation–associated molecule, CD83, and the costimulatory molecules, CD80 and CD86—markers of M1 macrophages [106].

#### 2.2 Involvement of the gastrointestinal tract and excretions/secretions (ES)

In addition to the tegument, other organs are also recognized as playing roles in controlling host immunity. For instance, the esophageal gland is capable of lysing ingested host immune cells [107]. Knocking down expression of the fork head-box transcription factor Sm-foxA in *S*. *mansoni* schistosomula leads to the generation of parasites that lack an esophageal gland [108]. These worms die in immunocompetent hosts, but survive in hosts lacking B cells, suggesting a heretofore unrecognized role for this gland in taming host immunity [108].

Blood ingested by schistosomes that passes the esophagus is broken down by proteases in the lumen of the parasite’s intestine. Hemoglobin digestion leads to the generation of a toxic biproduct, heme, and this is neutralized in the schistosome gut via its crystallization into the waste product **hemozoin** (**Hz**). Hz is periodically regurgitated into the host’s circulation where it can accumulate in the liver, being taken up by macrophages [109]. When incubated with murine bone marrow–derived macrophages in vitro, purified *S*. *mansoni* Hz alone is not observed to have any notable impact [109]. However, when administered at the same time as IL-4, Hz can potentiate the IL-4–induced expression of transcripts characteristic of AAMs; increased expression of the chitinase 3-like protein 3 (Ym1) and Arginase 1 (Arg1) are noted, along with greatly reduced expression of Resistin-like molecule alpha (Retnla), suggesting a potential immunomodulatory role for this schistosome metabolic waste [109].

*2*.*2*.*1*. *Released proteases and protease inhibitors*. Among the products released by worms in culture are proteases that could impact host immune function [110]. The cysteine protease **cathepsin B1** has been detected in some analyses of the secretomes of cultured adult schistosomes [111–113]. It has been shown that cathepsin B1 of *S mansoni* (**SmCB1**) can, in recombinant form, protect mice from the lethal effects of LPS by preventing the release of inflammatory mediators, NO, IL-6, TNF-α, and IL-12, from macrophages [114]. The protease blocks the MyD88-independent, TRIF-dependent signaling pathway of TLR4 and TLR3 [114].

**Sj-C** is a 101 amino acid cysteine protease inhibitor (**cystatin**) identified in *S*. *japonicum* that is expressed in the gut and tegument of adult worms (as well as in eggs) [115]. In addition, cystatins have been identified in some analyses of schistosome secretomes [111,112]. Recombinant Sj-C has been shown to suppress exogenous antigen presentation by murine spleen–derived DCs [116]. In addition, injecting mice that have chemically induced colitis with rSj-C significantly reduced inflammatory parameters and ameliorated the severity of disease; decreased IFN-γ levels and increased IL-4, IL-13, IL-10, and TGF-β levels in the colon were recorded [117]. In addition to cystatins, **serpins** (serine protease inhibitors) have also been found in some analyses of schistosome secretomes [111–113]. One *S*. *mansoni* serpin, designated ***Sm*KI-1**, binds to neutrophil elastase and impairs neutrophil migration and function in murine models of inflammatory diseases [118].

*2*.*2*.*2 Glycolytic enzymes*. One group of proteins found in most analyses of schistosome ES, and in the schistosome tegument (including at the host–parasite interface), are **glycolytic enzymes** such as **aldolase, enolase, glyceraldehyde 3-phosphate dehydrogenase** (**GAPDH**), and **triose phosphate isomerase** (**TPI**) [111,113,119–121]. While it is initially surprising to find these conserved, classically cytosolic enzymes in the extracellular environment, it is now clear that such proteins can have “moonlighting” functions that are unrelated to glycolysis [119]. For instance, extracellular forms of schistosome enolase and GAPDH can bind to mammalian plasminogen and promote its conversion to the active form—plasmin [122,123]. In other systems, several glycolytic enzymes have been shown capable of modulating immunity by, e.g., blocking complement action or impeding B cell function [119,123].

*2*.*2*.*3 Redox enzymes*. A second group of proteins that is consistently detected in schistosome ES as well as in the schistosome tegument are enzymatic drivers of redox reactions that likely act to control oxidative stress around the worms. Most lists of schistosome ES composition contain some combination of such proteins as **glutathione S transferases, thioredoxins, thioredoxin peroxidases (TPxs), peroxiredoxins, tryparedoxin peroxidases,** and **superoxide dismutases** [112,121,124,125]. These enzymes are considered central to the ability of the worms to neutralize phagocyte-generated, toxic oxygen- and nitrogen-based metabolites that could damage or kill larvae and adults. Knockdown of *S*. *japonicum* **peroxiredoxin-1** using RNAi renders the worms susceptible to H_2_O_2_ [126]. In addition, one member of this protein group—**SjTPx**—has, in recombinant form, been shown to induce M2 macrophage generation, with expression of anti-inflammatory effectors TGFβ, IL-10, and Arg1 as well as suppressed expression of proinflammatory cytokines such as TNF-α, IL-1β, IL-6, and iNOS following LPS stimulation [127]. Also, of note, a liver fluke (*Fasciola hepatica*) homolog of secreted schistosome **peroxiredoxin** can similarly induce the development of alternatively activated (M2) macrophages [128].

*2*.*2*.*4 Other released proteins*. Schistosome extracellular vesicle (EV) and excretion/secretion (ES) preparations also invariably contain one or more heat shock proteins (HSPs) [112,121,124,125]. HSPs are conserved, ubiquitously expressed proteins that play key roles in the cellular response to stress. Recombinant **SjHSP70** (like rSjTPx, mentioned above) can induce M2 macrophage generation as exemplified by its ability to stimulate macrophages to express TGFβ, IL-10, and Arg1 and suppress LPS-induced expression of TNF-α, IL-1β, IL-6, and iNOS [127].

Among the many protein components of worm ES are several that are homologs of known immunomodulators. For instance, *S*. *mansoni* **calreticulin** can be secreted [113], and calreticulin homologs in other parasitic worms are known to bind to complement C1q and inhibit activation of the classical complement pathway [129,130].

**Cyclophilins** are widely expressed peptidyl-prolyl *cis*-*trans* isomerases and are detected in some, but not all, analyses of the secretomes of cultured adult schistosomes [111,113]. Some cyclophilins can modulate BMDC function [131]. Enzymatically active, recombinant *S*. *mansoni* **cyclophilin A** can alter DC function and cytokine production in in vitro cell culture assays, leading to a DC-mediated preferential expansion of CD4+ Treg cells [113].

Finally, adult *S*. *mansoni* have been reported to release neuropeptides such as **corticotropin** (ACTH) and **β-endorphin** [132], and these also have the potential to regulate host immune cell function [133].

Fig 2 summarizes the major reported impacts of several components of intravascular stage schistosomes (schistosomula and adult worms) on a variety of immune cell types.

### 3. Eggs in tissues

Central to the generation of the characteristic polarized Th2 response seen following schistosome infection are parasite eggs, since a robust Th2 response, and IgE production, are only observed after egg deposition or following injection of schistosome eggs, or egg extracts, into naive animals [134–136]. Recall that during schistosome infection, many eggs do not exit the host but become trapped in local tissues inducing pathology. Unlike adult worms, schistosome eggs attract obvious immunological attention and become surrounded by an array of immune cells forming a structure known as a granuloma.

Several components released from schistosome eggs have been shown to drive Th2-biased immunity. A crude, soluble, schistosome egg fraction (designated SEA, for soluble egg antigen) was shown to be capable of inducing degranulation of isolated human basophils with the release of IL-4 and IL-13 (in addition to histamine and sulfidoleukotrienes) [136]. Crude, soluble extracts of schistosomula had no equivalent impact [136]. Given that IL-4 is a key cytokine responsible for biasing the immune reaction toward a Th2 phenotype and that IL-13 has a central role as a regulator of IgE synthesis, interest focused on uncovering the molecular component(s) of SEA that might mediate basophil reactivity leading to IL-4 and IL-13 release. Fractionation of SEA led to the identification of a bioactive protein designated “IL-4–inducing principle of schistosome eggs” or IPSE, described next [137].

#### 3.1 IPSE/α-1

IPSE has been found to be identical to a polypeptide previously described as alpha1 (α1), and the protein is now given the joint designation IPSE/α1. IPSE/α1 is a 134 amino acid homodimeric glycoprotein containing a 20 amino acid signal sequence and with a molecular mass of approximately 40 kDa [137]. The protein homodimerizes via an intermolecular disulfide bridge involving the C-terminal cysteine, C^132^ [138]. No sequences in protein databases exhibit substantial similarity to IPSE/α1 (except in other schistosome databases). Secondary structure predictions indicate a mainly β-sheet fold; NMR spectroscopy and crystallographic analyses place IPSE/α1 as a member of the βγ-crystallin protein family [139]. Native IPSE/α1 resolves by 2D gel electrophoresis into six 20 to 30 kDa spots, all with a pI of approximately 9.5 [140]. The biological significance of these variants is unknown.

Expression of IPSE/α1 at the level of both mRNA and protein is restricted to the egg stage; it is only present in mature eggs with synthesis being induced after egg laying [141]. Immunohistology reveals that the protein is enriched outside of the living parasite (the miracidium) but inside the eggshell and is secreted from this subshell area to the surrounding host tissue [142]. IPSE/α1 is secreted by mature eggs [140]; in infected animals, it can be detected in the vicinity of eggs and inside some cells around the egg [141]. The ability to detect both IPSE/α1 and basophils in granulomas around schistosome eggs in sections of liver and gut of infected mice indicates that the two can come into close contact with each other in vivo [143]. *S*. *mansoni*–infected mice and humans develop a pronounced antibody response against IPSE/α1 [141].

Sequence analysis reveals a nuclear localization signal (^125^PKRRRTY^131^) at the C terminus of IPSE/α1. Internalized recombinant IPSE/α1 can be detected in the nuclei of a variety of cell types including Huh7 (human liver carcinoma), U2OS (human osteosarcoma), and CHO (Chinese hamster ovary) cells [144]. Work with IPSE/α1 homologs from *S*. *haematobium* (designated H-IPSE) confirm that C-terminal nuclear localization signals are functional and essential for the translocation of the protein into the nuclei of host cells where the protein may bind DNA [145]. IPSE/α1 also carries glycans at 2 N-glycosylation sites, of which one is not always occupied. At these sites, the protein contains core difucosylated diantennary glycans that possess one or more Lewis X (LeX) motifs (Galβ1–4(Fucα1–3)GlcNAc) [146]. However, glycosylation is not required to promote IL-4 production by basophils since nonglycosylated, recombinant IPSE/α1 produced in *E*. *coli* displays similar activity as the native glycoprotein [137].

When IPSE/α1 is incubated with cultured murine primary hepatocytes, cell injury is detected in dose response (as measured by increased alanine transaminase (ALT) activity), leading to the conclusion that IPSE/α1 can be hepatotoxic [147]. Injection of recombinant IPSE/α1 into non-schistosome–infected reporter mice rapidly induces dose-dependent IL-4 production by basophils in the liver [148]. However, total splenocytes recovered from mice injected with rIPSE/α1 evoked a mixed Th1/Th2 cytokine response following rIPSE/α1 restimulation in vitro, generating appreciable levels of IFN-γ, IL-5, and IL-13 and lower levels of IL-4 and TNF-α [149]. IPSE/α1 has been found to be a general immunoglobulin-binding protein with highest affinity for IgE [139]. The protein is thought to exert its impact on basophils by binding to, but not cross-linking, receptor-bound IgE [139].

The ability of IPSE/α1 to drive IL-4 and IL-13 production in basophils may also be important in the generation of anti-inflammatory, alternatively activated (M2) macrophages; as noted earlier, this is a feature of schistosome infection [21]. LPS-activated monocytes cultured in the presence of basophils that had been exposed to IPSE/α1 elevate expression of CD206 (mannose receptor) and CD209 (DC-SIGN) [143]. Additionally, IL-4/IL-13 produced by IPSE/α1-stimulated basophils can inhibit the release of the proinflammatory cytokines IL-1b, IL-6, and TNF-α from LPS-activated monocytes [143].

As noted earlier, infection with schistosomes also induces Breg cell generation in mice and in humans [150–152], and IPSE/α1 may play a role here, too. Injecting mice with whole *S*. *mansoni* eggs or SEA or IPSE/α1, in the absence of a natural worm infection, has been found to be sufficient, by itself, to drive splenic Breg cell development [153]. Only marginal zone B cells of the spleen, but not follicular B cells, are responsive to the injected material [153]. These cells are observed to bind fluorescently labeled SEA or IPSE/α1 in a dose-dependent manner following in vitro culture. SEA- or IPSE/α1-stimulated B cells secreted elevated levels of IL-10 (but not IL-6) compared to controls and were capable of driving CD25+Foxp3+ Treg cell development during B cell/T cell coculture [153]. It has been hypothesized that the immunoglobulin-binding capacity of IPSE/α1 allows it to connect to B cells via surface-exposed IgG, or binding may occur via the B cell receptor [153]. Since SEA depleted of IPSE/α1 is still as efficient as total SEA in inducing IL-10 secretion by splenic B cells, this shows that SEA components in addition to IPSE/α1 can activate these cells [153].

Given the ability of IPSE/α1 to modulate host immunity and dampen inflammation, the protein is currently being tested therapeutically. Pig fibroblasts, engineered to constitutively express IPSE/α1, were subcutaneously injected into mice, and this polarized the splenic lymphocyte immune response during xenograft rejection in the Th2 direction while suppressing the Th1 response [154]. Injection of IPSE/α1 either systemically or directly into the bladder wall was reported to be therapeutically useful in a murine model of hemorrhagic cystitis [155,156]. Treatment attenuated chemically induced, and likely pathological, increases in bladder wet weight in an IL-4–dependent fashion [155,156].

#### 3.2 Omega-1 (ω1)

A second protein that is expressed in schistosome eggs and in egg ES, and that could play a pivotal role in directing host immunity, is omega-1 (ω1)—a 225 amino acid monomeric glycoprotein with a 23 amino acid predicted signal peptide. [157,158]. The protein has been reported to drive human MoDCs to prime highly Th2-polarized responses from naïve human CD4+ T cells in vitro [159]. Omega-1 exposure to DCs also significantly impairs LPS-induced up-regulation of CD83 and CD86 surface expression, as well as IL-12 release (as does SEA) [159]. When such DCs are subsequently cocultured with naïve CD4+ T cells, the T cells become Th2 polarized, as revealed by strong intracellular IL-4 staining [159]. Injecting ω1 into mice similarly induced a marked Th2 response and the production of IL-4 [159].

These findings involving human DCs and ω1 are replicated in mice; SEA- or ω1-conditioned murine BMDCs prime Th2 responses in vitro and also upon transfer into naïve mice [160]. SEA chemically depleted of ω1 does not trigger Th2 polarization [160]. While SEA or ω1 treatment did not significantly alter murine DC viability, this did lead to overt changes in morphology such that treated cells were less adherent to plastic in culture [160].

Omega 1 has 2 fully occupied N-glycosylation sites (N^71^, N^176^) [161,162]. Both sites display similar glycan heterogeneity, but with different relative abundances of the individual glycans. All glycans on ω1 are of a diantennary complex type [162]. LeX is the major antenna motif, but other terminal glycan elements are also present [162]. Omega-1 also contains a pair of conserved cysteine residues (Cys^72^, Cys^117^) considered important to maintain active conformation [161]. Native ω1 in egg ES resolves by 2-DE as 3 spots, two of which have similar pIs (9.5) but are of slightly different mass (30 and 35 kDa); a third, smaller isoform (approximately 9 kDa) has a lower pI (9.0) and may be a splice variant or a product of proteolysis [140].

Omega-1 expression was restricted to the *S*. *mansoni* egg with the fully developed egg secreting considerably more protein than the immature egg [163]. No mRNA for ω1 was detected in adult females or miracidia [161]. Omega-1 is a natural immunogen and provokes a strong antibody response in *S*. *mansoni*–infected rodents and humans [157].

Database comparisons revealed that ω1 shares similarity with members of the T2 Ribonuclease enzyme family and possesses conserved amino acid sequence blocks (CAS-1 and CAS-2) that contain residues critical for ribonuclease catalytic activity [161]. Subsequently, negative staining zymography showed that ω1 is a functional ribonuclease [161].

Experiments employing site-directed mutagenesis of ω1 revealed that both the glycosylation and the RNase activity of the protein are essential for its Th2-inducing activity both in vitro and in vivo [164]. This work reveals that ω1 binds to DCs and is internalized via its glycans by the mannose receptor (MR, CD206); the protein can be detected throughout the cell after 1 hour’s incubation [164]. Preferential breakdown of 28S ribosomal RNA is first detected, with breakdown of 18S rRNA becoming apparent at later time points. Concurrently, a progressive reduction in mRNAs encoding several unrelated genes is observed [164]. Thus, the observed inhibition in protein synthesis in ω1-treated DCs is a combined effect of degradation of mRNA transcripts and interference with ribosomal integrity caused by rRNA cleavage. However, analysis of DCs 32 hours after exposure to ω1 (or SEA) reveals that RNA degradation is not uniform; there is substantially increased expression of certain genes, e.g., ribosomal protein P2 and synaptic vesicle amine transferase-1 [165]. Cumulatively, these changes are said to lead to reduced DC–T cell interaction, a setting that favors Th2 polarization [160].

Another immunological impact of ω1 is its ability (like that of complete SEA) to enhance IL-1β secretion by peritoneal macrophages that have been also stimulated with TLR2 ligand (synthetic triacylated lipopeptide Pam3CysSerLys4) [166]. Blocking the C-type lectin receptor Dectin-1 (but not the Dectin-2 receptor or MR) almost completely abrogated the effect, which was found to be also dependent on caspase-8 and ASC (apoptosis-associated speck-like protein containing a caspase recruitment domain) [166]. In this work, secretion of other IL-1 family members, IL-18 and IL-33, was not seen. SEA that has been depleted of ω1 does not enhance IL-1β secretion, showing that ω1 is the principal component in SEA that can mediate this effect [166].

The ability of ω1 to impinge on host immunology has resulted in its being employed therapeutically in a mouse model of obesity [167]. Treatment of obese mice with ω1 induced a potent Th2 cell response in the adipose tissue and with the release of IL-33 from adipocytes. Cells within the adipose tissue, and in the peritoneal cavity, released type 2 cytokines, resulting in the local accumulation of innate type 2 lymphoid cells in the epididymal white adipose tissue. These changes led to the downstream polarization of classically activated macrophages in the adipose tissue to an alternatively activated (M2) macrophage phenotype, resulting in a favorable therapeutic outcome—stabilization of glucose homeostasis in the obese mice [167].

In the mouse model of autoimmune type 1 diabetes, it was shown that either live *S*. *mansoni* infection or a parasite-derived soluble antigen preparation confers protection against diabetes in nonobese diabetic (NOD) mice [168]. This has spurred interest in the interaction of individual egg components such as ω1 in this experimental system. It has been found that immature BMDCs from NOD mice, costimulated with LPS and ω1, secrete significantly higher amounts of TGF-β as well as IL-1β compared to those exposed to LPS alone [169]. Further, these cells expressed higher mRNA levels for Raldh2 (retinaldehyde dehydrogenase 2). When these DCs are cocultured with naïve CD4+T cells, increased expression of the Treg cell marker Foxp3 is noted. Omega-1 is reported not to have any direct effect by itself on T cells [169], and its ability to drive Treg generation represents a novel function of this protein. Following a single subcutaneous injection of ω1 in the footpad of mice, both the percentage and the number of Foxp3+CD4+T cells in the draining lymph nodes of wild-type C57Bl/6 or NOD mice increase. Additionally, the Th2 cytokines IL-4 and IL-13 are significantly increased in CD4+T cells from these mice and expression of IFN-γ and IL-17A are up-regulated [169]. In other work, treatment of obese mice with recombinant ω1 decreased body fat mass and improved both systemic insulin sensitivity and glucose tolerance. This effect was associated with an increase in white adipose tissue Th2 cells, eosinophils, and M2 macrophages. Of note, these metabolic effects were still observed following administration of ω1 to obese mice with impaired type 2 immunity (STAT6-knockout strain). This indicates that the metabolic effects observed here were independent of type 2 immune responses [170].

In *S*. *japonicum*, the closest homolog to *S*. *mansoni*’s ω1 is SjCP1412; however, there is only 30% amino acid identity between the 2 proteins [171]. SjCP1412 has been confirmed as an RNase T2 family protein with RNase activity. Like ω1, SjCP1412 is released only from parasite eggs, not from cercariae or adult worms. Stimulating macrophages (RAW264.7 cells) with recombinant SjCP1412 increased expression of CD206, Arg-1, and IL-10, (all markers of alternatively activated (M2) macrophage differentiation) [171]. Stimulating DCs with rSjCP1412 failed to induce their maturation and inhibited LPS-stimulated DC maturation. Injecting mice with rSjCP1412 lowered serum IFN-γ levels but increased serum IL-4 and TGF-β levels as well as splenic CD4+CD25+Foxp3+ Treg cell numbers [171]. In sum, the effects of SjCP1412 broadly mimic those recorded for ω1 from *S*. *mansoni*.

In order to explore the importance of ω1 for parasite egg biology, a lentivirus-based transduction system was used to deliver microRNA (miRNA)-encoding short hairpin RNAs to suppress ω1 gene expression in isolated *S*. *mansoni* eggs. This led to a 50% to 60% reduction in ω1 transcript levels by day 3 after viral transduction [172]. Such ω1 gene–suppressed eggs, when injected into mice, provoked significantly smaller granulomas in the lungs when compared with controls. In addition, there was a reduction in the numbers of DCs, T-helper cells, and B cells, as well as macrophages in the lungs of mice injected with these ω1 knockdown eggs [172].

In another approach, CRISPR/Cas technology was used to knock out the ω1 gene in *S*. *mansoni* eggs. Sequencing analysis revealed that this method yielded less than 5% efficiency in gene editing [173]. Remarkably, this was accompanied by a >80% reduction in ω1 gene expression. Extracts of such ω1-mutated eggs exhibited reduced RNase activity as well as a reduced ability to polarize Th2 cytokine responses in cocultured macrophages and T cells; significantly less of the Th2 cytokines IL-4 and IL-5 could be detected in these cell coculture supernatants compared to supernatants from cocultures of cells incubated with wild-type egg extracts. Furthermore, following introduction of eggs into the tail veins of mice, the volume of pulmonary, circumoval granulomas around the ω1-deficient eggs was significantly reduced compared to those provoked by wild-type eggs [173].

#### 3.3 Kappa-5

A third identified component of *S*. *mansoni* SEA is **kappa-5**. This is a 300 amino acid glycoprotein that exists as a dimer of 2 approximately 50-kDa monomers [174]. Kappa-5 homologs are found only in other schistosome species. Kappa-5 has 4 fully occupied N-glycosylation sites carrying unique triantennary glycans composed of a difucosylated and xylosylated core region and immunogenic GalNAc1–4GlcNAc (LDN) termini [162]. The protein immunolocalizes to the subshell area of the egg and is expressed in miracidia. In addition to eliciting IgG antibodies, kappa-5 is also the target of a pronounced IgE isotype antibody response in the human host [174]. Kappa-5 has been suggested to be involved in the regulation of fibrosis, possibly having a modulatory effect on macrophages that control collagen synthesis [172].

#### 3.4 SmCKBP—Chemokine binding glycoprotein

An additional immunoregulatory molecule that is known to be secreted from *S*. *mansoni* eggs into host tissues is the approximately 36-kDa chemokine binding glycoprotein SmCKBP. This protein can bind to selected chemokines and inhibit their interaction with their respective cellular receptors [175]. The protein is detected within the parasite egg and within the granuloma surrounding intact eggs in infected mice but is not found at any other stage of the life cycle, in older granulomas or in dead eggs. Recombinant SmCKBP binds to and neutralizes the impact of IL-8, CCL2, CCL3, CCL5, and CXCL1 and can inhibit neutrophil, but not eosinophil, migration [175]. Deglycosylated SmCKBP retains bioactivity. The protein helps determine the cellular makeup within granulomas because blocking SmCKBP in vivo yields larger egg-induced granulomas that have increased numbers of neutrophils and macrophages [175]. SmCKBP shares no appreciable amino acid sequence similarity with known viral chemokine binding proteins or with any mammalian protein. When administered systemically, rSmCKBP is an efficacious inhibitor of chemokine-induced pulmonary inflammatory responses in murine models [175].

#### 3.5 SmHMGB1—High mobility group protein 1

High mobility group box 1 protein 1 (HMGB1) is an important nuclear protein that helps regulate transcription. In addition, macrophages and DCs can secrete the protein, which is proinflammatory. A 176 amino acid HMGB1 homolog from *S*. *mansoni* (SmHMGB1) can be detected in egg stage ES [176]. In recombinant form, the impact of this protein is counter to that of several of the other eggs proteins discussed here; it can induce up-regulation of proinflammatory cytokine (TNF-α, IL-1Rα, IL-2Rα, IL-6, IL-13, IL-13Rα1, IL-15, and MIP-1α) gene expression in mouse peritoneal macrophages [176].

#### 3.6. The egg and the granuloma

It seems likely that several of the egg secreted proteins discussed here, and others, through their interactions with both immune and nonimmune cells (such as epithelial cells and fibroblasts), also help facilitate egg translocation through host tissues and into the lumen of the gut or bladder. From here, the mature eggs can most easily exit the body of the host to continue the parasite’s life cycle. For instance, **SjE16.7** is a 145 amino acid, egg-specific, secreted protein that has been shown to act as a neutrophil chemoattractant that has been hypothesized to help facilitate egg excretion [177].

Egg excretion is considered an immune-dependent process as illustrated by significantly fewer eggs passed by infected, T cell–deficient [178,179], or nude mice [180]. In addition, severe combined immunodeficient (SCID) mice are almost incapable of excreting parasite eggs in the early weeks of oviposition [181]. Thus, eggs laid in the vasculature must induce regulated Th2-biased inflammation in order to both facilitate extravasation through the intestinal or bladder wall as well as protect surrounding host tissues from egg-derived toxins (reviewed in [182,183]). How the molecular cross talk between egg molecules and the diverse cells of the granuloma assists in the directional movement of a parasite egg from the endothelium toward the epithelium is not known.

#### 3.7 Transforming growth factor β (TGF-β) family members

While schistosomes have been reported to express proteins belonging to the transforming growth factor-β (TGF-β) superfamily [184,185], there is no evidence that these schistosome proteins have any immunomodulatory impact. The TGF-β family is evolutionarily ancient, and most helminth genomes, including free-living helminths, encode TGF-β family members. Thus, schistosome TGF-β family members are proposed to largely play roles in embryogenesis, oviposition, and parasite reproductive biology [184,185]. In contrast, TGF-β homologs from other trematodes may have an immunological impact. For instance, the *Fasciola hepatica* TGF-like molecule FhTLM can ligate the mammalian TGF-β receptor and promote IL-10 and arginase expression in macrophages [186].

**Fig 3:**
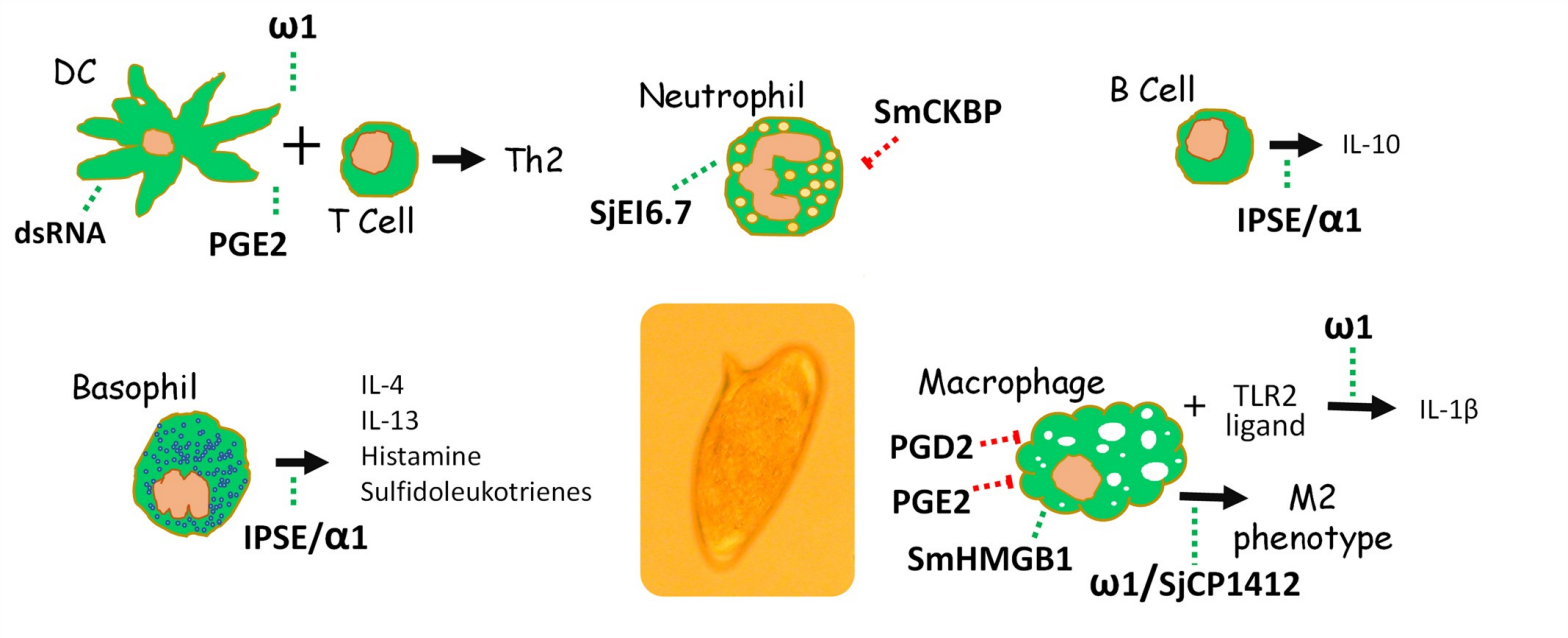
The impact of molecules (bold text) produced by schistosome eggs on the host immune cells indicated. The green dashed lines indicate stimulatory effects, while red dashed lines indicate inhibitory effects. An image of an *S*. *mansoni* egg is seen at center. See text for details regarding the impact of individual schistosome molecules on specific cell types. DC, dendritic cell; dsRNA, double-strand RNA; IPSE/α1, IL-4–inducing principle of schistosome eggs/alpha 1; PGE2, prostaglandin E2; PGD2, prostaglandin D2; SmCKBP, *S*. *mansoni* chemokine binding protein; SmHMGB1, *S*. *mansoni* high mobility group box 1 protein 1.

Fig 3 summarizes the major reported impacts of several ES components of schistosome eggs on a variety of immune cell types.

Beyond the impact of proteins on immune cell function, it has been demonstrated that *S*. *mansoni* isolated egg-derived **double-stranded RNA** (or whole eggs) can activate murine BMDCs in a TLR3-dependent manner, leading to increased production of IL-12p40 and TNF-α [187]. In addition, schistosome lipids, fatty acids, and carbohydrates may also exert considerable impact on host immunity, as outlined next.

### 4 Immunomodulatory schistosome lipids and fatty acids

#### 4.1 Lyso-phosphatidylserine (Lyso-PS)

To explore a possible involvement of schistosome lipids in immunomodulation, human MoDCs were exposed in vitro to lipid fractions derived from *S*. *mansoni* eggs or adult worms. The lipid fraction containing phosphatidylserine (PS) was found to polarize the maturation of these DCs such that, when cocultured with naïve T cells, they induced Th2 skewing (i.e., the generation of T cells enriched for intracellular IL-4 staining) as well as the generation of IL-10 producing Tregs [188]. It was shown that activation of TLR2 on DCs was essential for the development of the IL-10 producing T cells. Furthermore, fractionation of the PS mixture revealed that a unique schistosome lyso-phosphatidylserine (lyso-PS) was the TLR2-activating moiety [188]. The lyso-PS of eggs and adult worms was not identical: The fatty acids present in lyso-PS from adult schistosomes were 20:1 and 22:4, whereas the most abundant lyso-PS species in eggs were 26:1, 24:0, 18:0, 26:2, and 26:0 [188]. These differences may explain the generally greater potency of the egg PS fraction compared to the adult fraction [188]. Synthetic lyso-PS (16:0) was unable to exert an impact on DC polarization or on TLR activation, suggesting that the structure of the acyl chain found in schistosome lyso-PS is critical for its biological activity [188].

These findings of schistosome lipid immunoreactivity are intriguing since it has been known for some time that the species composition of the phospholipids in the adult worms’ tegumental membranes differ substantially from those of whole adult worms as well as from those of blood cells of the host [189,190]. Specifically, the tegumental membranes are enriched in lysophospholipids, predominantly eicosenoic acid (20:1)-containing lyso-PS and lyso-phosphatidylethanolamine species [191]. Perhaps immunostimulatory tegumental lyso-PS acts on host sentinel DCs in vivo. These, upon migrating to draining lymph nodes, might then activate naïve T cells, polarizing T cell development toward a Th2 and Treg phenotype, both features characteristic of immune responses in chronic schistosome infections.

A caveat here is that it is not known to what extent tegumental lipids are exposed to host cells. Schistosomes do produce and release EVs (described later); therefore, it is possible that EV lipids mediate the effects noted [192]. However, secretion of lyso-phospholipid (in EVs or otherwise) by schistosomes has not been reported; none of the schistosome-specific lyso-phospholipid or phospholipid species was seen in medium in which schistosomes were incubated for 2 hours [191]. The possible excretion or secretion of tegument-specific lyso-phospholipids was also investigated in vivo by analysis of total phospholipids present in blood plasma (taken 45 to 48 days postinfection) of infected hamsters (each containing approximately 100 adult worms). None of the schistosome-specific or tegument-specific lyso-PS species was increased in the plasma derived from schistosome-infected hamsters compared with noninfected hamsters [191].

#### 4.2 Lysophosphatidylcholine (LPC)

Exposing peritoneal, or bone marrow–derived, murine macrophages to an adult *S*. *mansoni* lipid extract, or to purified schistosome lysophosphatidylcholine (LPC), induced an AAM (M2) profile, as seen by increased expression of arginase-1, mannose receptor 1 (MR1), chitinase 3-like 3 (Ym1), TGF-β, 24 hours after stimulation; production of IL-10 and PGE2 was also observed [193]. Activation occurred in a PPAR-gamma (peroxisome proliferator activated receptor-gamma)-dependent manner. Together, LPC 16:0 and LPC 18:0 comprise >94% of the schistosome LPC species isolated [193]. Synthetic LPC16:0, but not synthetic LPC18:0, was found to induce Arg-1 expression in the macrophages, indicating that the size of the fatty acid chain influences the outcome [193]. In addition, a schistosome total lipid extract, or purified schistosome LPC, can induce TGF-β secretion from isolated human eosinophils [194].

#### 4.3 Eicosanoids

Arachidonic acid is a polyunsaturated omega-6 fatty acid that is the precursor to several biologically important signaling molecules collectively called eicosanoids. Enzyme families that are important in eicosanoid biosynthesis include the cyclooxygenases (Coxs, that yield, e.g., prostaglandins) and the lipoxygenases (Loxs, that yield, e.g., hydroxyeicosatetraenoic acids, HETEs). Adult *S*. *mansoni* extracts display lipoxygenase, but not cyclooxygenase, activity [195]. The Lox activity catalyzes the formation of a 15-HETE-like species, and the activity is calcium independent and blocked by inhibitors of mammalian and plant Lox enzymes. Despite an inability to detect Cox enzyme activity in schistosome extracts [195], adult schistosomes and schistosomula—when incubated with linoleic acid (a metabolic precursor to arachidonic acid)—secrete eicosanoids belonging to both the Lox and the Cox classes. These include 15-HETE and 5-HETE as well as leukotriene (LT)B4 and PGE2. Schistosomula and adult females also produce LTC4, while adult males do not [196]. Analysis of the collection of lipids excreted or secreted by approximately 200 adult male and female *S*. *mansoni* after incubation for 48 hours in 20 mls of culture medium (and here lacking added linoleic acid) also revealed relatively high levels of 15-HETE in addition to 12-HETE, PGE2, and PGD2 [197]. How might the secretion of these metabolites benefit schistosomes? 15-HETE is known to be a ligand for PPARs [198]; since activation of these receptors on immune cells can lead to attenuated inflammatory effector responses, it is possible that schistosome release of 15-HETE contributes in this way to immune suppression.

Both PGE2 and PGD2 can have proinflammatory or anti-inflammatory effects depending on circumstances [199]. Of interest in the context of the Th2 polarization that accompanies schistosome infection is the ability of PGE2 to condition DCs to prime Th2 responses [200]. Additionally, PGE2 selectively suppresses effector functions of macrophages and neutrophils as well as type-1 immunity, while promoting Th2, Th17, and Treg responses—outcomes considered to support schistosome survival [199]. In addition to producing their own PGE2, the parasites can induce PGE2 (as well as IL-10) production in human and mouse keratinocytes, and this could help down-regulate damaging host immune responses in the skin [46].

Regarding PGD2, this prostaglandin has been linked to impeding the host’s immune response against cercariae [201], possibly through its ability to block migration of Langerhans cells in the skin [202]. PGD2, acting through the G-protein–coupled receptor DP1, has also been shown to trigger the release of TGF-β from isolated human eosinophils [194]. In addition, as noted earlier, schistosome LPC, acting through TLR2, can also induce TGF-β secretion from eosinophils [194]. These findings are notable, given that eosinophilia is a hallmark of schistosome infection and that TGF-β can inhibit T cell, B cell, and natural killer (NK) cell proliferation [203]. TGF-β, in turn, has been shown to stimulate murine hepatic stellate cells (recovered from schistosome granulomas) to synthesize and secrete PGD2 [204]. PGD2 is known to inhibit the functions of platelet, neutrophils, basophils, and DCs [205]. Cultured *S*. *mansoni* eggs themselves have been shown to release PGD2, as well as other potentially immunoregulatory lipids, such as PGE2 and 8-HETE [197]. Several isomers of PGE2 are also detected in relatively high amounts in schistosome egg ES [197]. Of note, PGE2 (or LPC) can induce secretion of additional eicosanoids—eoxin C4 (EXC4) and LTC4 (in addition to the abovementioned secretion of TGF-β) from isolated human eosinophils [194].

In sum, there is tremendous potential for eicosanoids produced and released by different schistosome life stages, as well as host-generated eicosanoids that are induced by the parasites, to engage in immunomodulation, and much work is required to disentangle the impacts of these various and powerful signaling metabolites in vivo.

### 5. Immunomodulatory schistosome glycans

#### 5.1 LNFPIII, LNnT, LDNF

Schistosome glycans lack sialic acid—a common terminal sugar that is found in glycoproteins and glycolipids of vertebrate cells [206]. In addition, schistosome N-linked and O-linked glycans often contain poly-fucose and xylose—modifications not found on vertebrate glycans [206]. It has long been recognized that some schistosome carbohydrates can modulate immune cell function [207]. For example, lacto-*N*-fucopentaose III (LNFPIII) is an immunomodulatory glycan that contains the LeX trisaccharide (Galβ(1–4) [Fucα(1–3)] GlcNAc) and that is expressed widely in schistosomes but is also found on human tissues [208]. Injection of LNFPIII can induce the expansion of peritoneal macrophages that are capable of suppressing naïve T cell proliferation in vitro [209]. The macrophages exhibit rapid up-regulation of Arg1 and Ym1, indicative of (M2) alternative activation [210]. Adoptive transfer of these macrophages induces naïve T cells to produce high levels of IL-10 and IL-13 [210]. LNFPIII was also reported to induce transient activation of NF-κB in murine BMDCs via TLR4 activation [211]. This is suggested to lead to the maturation of DC2s that drive Th2 responses.

In addition, LNFPIII, and related glycans, induce proliferation of splenic B cells of schistosome-infected or naïve mice [212]. Of the glycans tested, LNFPIII is the only one that induces isolated B cell–enriched spleen cells from infected animals to produce increased amounts of IL-10 and PGE2 (but not IL-4) [212]. These released biomolecules could help down-regulate Th-1 cells and support expansion of Th-2 cell populations following schistosome infection [212]. Indeed, LNFPIII has been shown to act as an adjuvant to promote type 2 immune responses [213]. LNFPIII can also induce proliferation of PBMCs isolated from schistosome-infected individuals [214]. Further, LNFPIII induces secretion of IL-10 by PBMCs recovered from individuals with low to moderate schistosome infection (meaning they excrete ≤400 parasite eggs per gram feces) but does not induce IL-10 secretion in PBMCs from highly infected subjects (i.e., those excreting >400 egg/gm feces) [214].

LeX, on LNFPIII or otherwise, is abundant and widely expressed in intravascular stage schistosomes, including at the parasite surface [215] and in eggs and egg ES [216]. Indeed, LeX is found on 2 potent immunomodulatory egg ES glycoproteins, IPSE/α1 and ω1, discussed earlier, and glycosylation is key for the immunomodulatory ability of ω1 but not IPSE/α1. LeX expression is common among different schistosome species but is absent in some other parasitic helminths like the trematode *Fasciola hepatica* and the nematode *Dirofilaria immitis* [217]. Of note, serum from schistosome-infected rodents [218], monkeys [219], and humans [217,219,220] all contain anti-LeX antibodies.

Another glycan expressed in schistosomes (and in humans) is LNnT (lacto-N-neotetraose; i.e., LNFPIII without fucose). Injecting LNnT intraperitoneally induces the recruitment of a macrophage population that produces IL-10 and TGF-β and suppresses naïve T cell proliferation [221]. These findings suggest that schistosome glycans such as LNFPIII and LNnT play roles in driving immunological events in a Th2 direction following schistosome infection.

Finally, some schistosome glycans are known ligands for C-type lectin receptors (CLRs), such as DC-SIGN expressed on DCs [222]. For example, LeX and another common schistosome glycan LDNF (GalNAcβ1–4[Fucα1–3]GlcNAc-) recognize DC-SIGN [222]. In addition, schistosome adult and egg glycoproteins carry a range of high-mannose-type and truncated oligomannosidic-type N-glycans [223,224], which likely could also bind DC-SIGN as well as CD206 that is expressed on macrophages and DCs. Conceivably such interactions could impact immune regulation in schistosome-infected individuals.

### 6. Extracellular vesicles (EVs)

Schistosomes are known to release several classes of lipid membrane–enclosed bodies collectively called extracellular vesicles (EVs) [225,226]. EVs are secreted by many cells; they carry bioactive molecules that are important in intercellular signaling. Different schistosome life stages have been reported to generate and release EVs with overlapping but distinct composition [125]. Not surprisingly, the protein composition of schistosome EVs and that of schistosome ES exhibit considerable overlap. A large proportion of the proteins recovered from both EVs and ES are metabolic enzymes, particularly those associated with glycolysis such as GAPDH, aldolase, and enolase [111,113,119–121,125,227]. Both preparations invariably contain enzymes that help control oxidative stress such as GST, thioredoxin, and thioredoxin peroxidase, and both contain several proteases [111,113,119–121,125,227].

Work with EVs also revealed the presence therein of **miRNAs**; these are small molecules of approximately 23 nucleotides that regulate gene expression by binding to target mRNAs, often blocking mRNA translation. Over 200 different miRNAs have been described in schistosome EV preparations, and schistosome miRNAs have also been found outside of EVs [228]. A second population of small RNAs derived from tRNAs (and designated tsRNAs) have also been described in schistosome EV preparations; the biological role of these RNAs is not known [228].

It has been shown that EVs from *S*. *japonicum* can be taken in by murine liver cells in vitro where they transfer their cargo miRNAs [227]. Exposure of macrophage RAW264.7 cells to *S*. *japonicum* EVs promoted M1-type polarization with increased production of proinflammatory mediators such as TNF-α, iNOS, and IL-12 [226]. However, equivalent exposure of bone marrow–derived murine macrophages to *S*. *mansoni* EVs had no detectable impact on the cells [125].

Exposing murine peripheral immune cells to labeled *S*. *japonicum* EVs leads to EV uptake predominantly by monocytes, followed by T cells, B cells, and NK cells [229]. RAW264.7 cells in vitro similarly take up such EVs; cargo miRNAs are transferred, and gene expression is altered. RAW264.7 cells treated with EVs proliferate (but heat-treated EVs exert no such effect) [229]. One miRNA (**miR-125b**) is highly enriched in *S*. *japonicum* EVs, accounting for 64% of the total. Transfection of RAW cells with miR-125b alone leads to the down-regulation of predicted target mRNAs, including those encoding the immune cell function regulator protein S1 (Pros1) and F11r. Significantly elevated levels of TNF-α are also recorded in the cell culture medium. Finally, following EV injection into mice, proliferation of monocytes and increased TNF-α production are noted [229].

*S*. *mansoni* EVs were shown to be internalized by murine T cells in vitro [230]. Schistosome miRNAs (notably **miR-10**, **miR-125**, and **Bantam**) were detected inside the EVs as well as within the T cells. Exposure of human Jurkat T cells to either live *S*. *mansoni* adults or to plasmid expressing miR-10 resulted in reduced expression of MAP3K7, a serine/threonine kinase that is involved in the activation of the key immune regulator NF-kB [230].

*S*. *mansoni* EVs have also been shown to be taken in not just by immune cells (human THP-1 monocytes) but also by cultured human umbilical vein endothelial cells (HUVECs) resulting in changes in gene expression [231]. In EV-exposed HUVEC cells, altered expression of genes that coordinate immune cell regulation, proliferation, differentiation, and signaling, such as IL-6 and CXCL2 were recorded [231].

Labeled *S*. *japonicum* EVs, injected into mice, are taken in mostly by monocytes [229]. Furthermore, miRNAs can be detected in EVs isolated from the sera of *S*. *mansoni*–infected patients [232]. Finally, schistosome miRNAs miR-10 and Bantam were detected in T cells isolated from Peyer’s patches and mesenteric lymph nodes of schistosome-infected mice [230].

Moving beyond proteins and miRNAs, it has been suggested that EVs released by schistosomes are also the source of immunomodulatory lipids and fatty acids, as described earlier [192]. Overall, it is clear that multiple components of the EVs released by schistosomes—protein, miRNA, and lipid could all play substantial roles in shaping host immunity in infected animals.

## Conclusions

**Fig 4:**
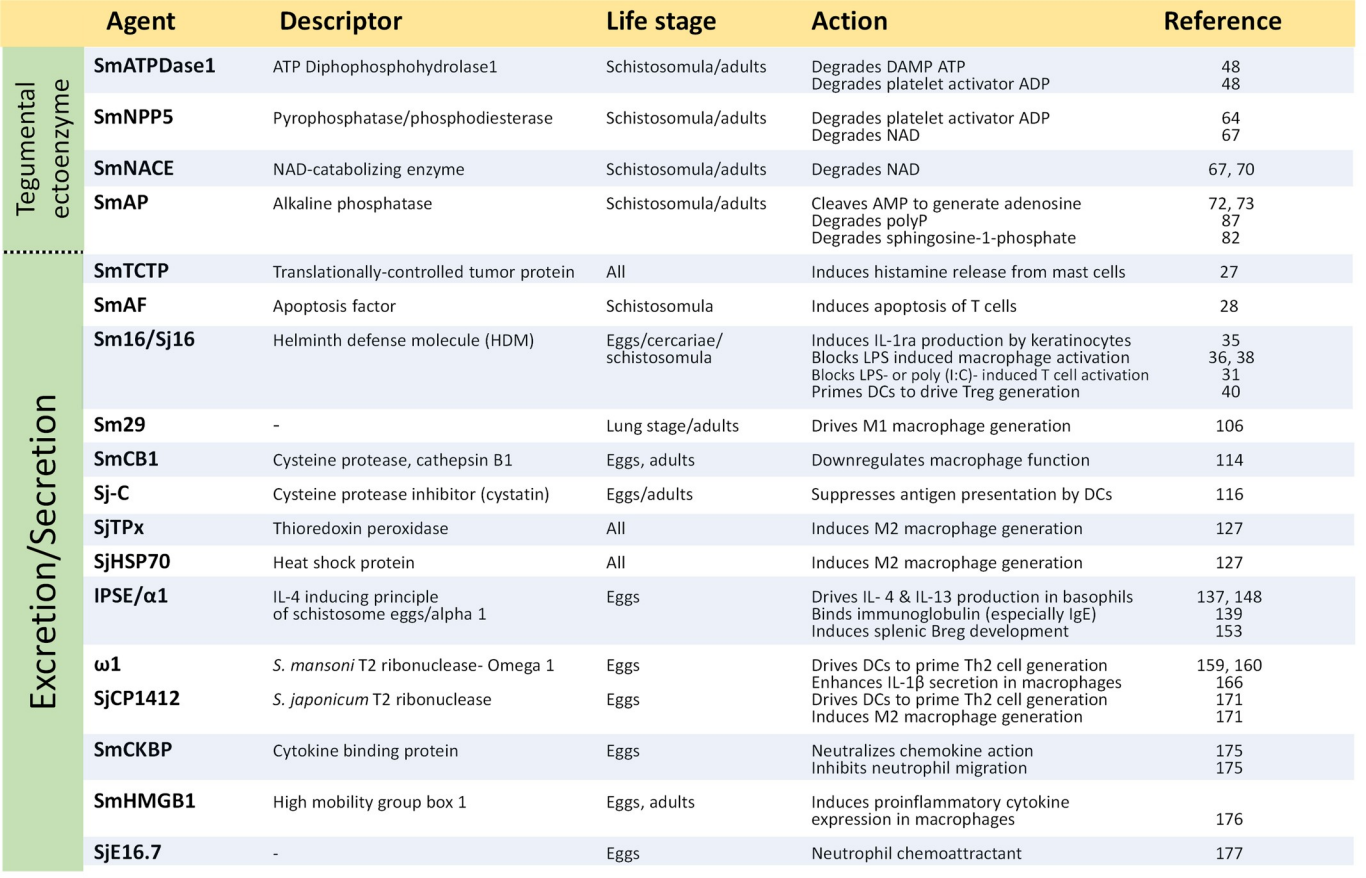
List of immunomodulatory schistosome proteins and their major effects. ADP, adenosine diphosphate; AMP, adenosine monophosphate; ATP, adenosine triphosphate; Breg, regulatory B cell; DC, dendritic cell; HDM, helminth defense molecule; IL-1ra, IL-1 receptor antagonist a; LPS, lipopolysaccharide; NAD, nicotinamide adenine dinucleotide; polyP, polyphosphate; SmAF, *S*. *mansoni* apoptosis factor; SmTCTP, *S*. *mansoni* translationally controlled tumor protein; Treg, regulatory T cell.

**Fig 5:**
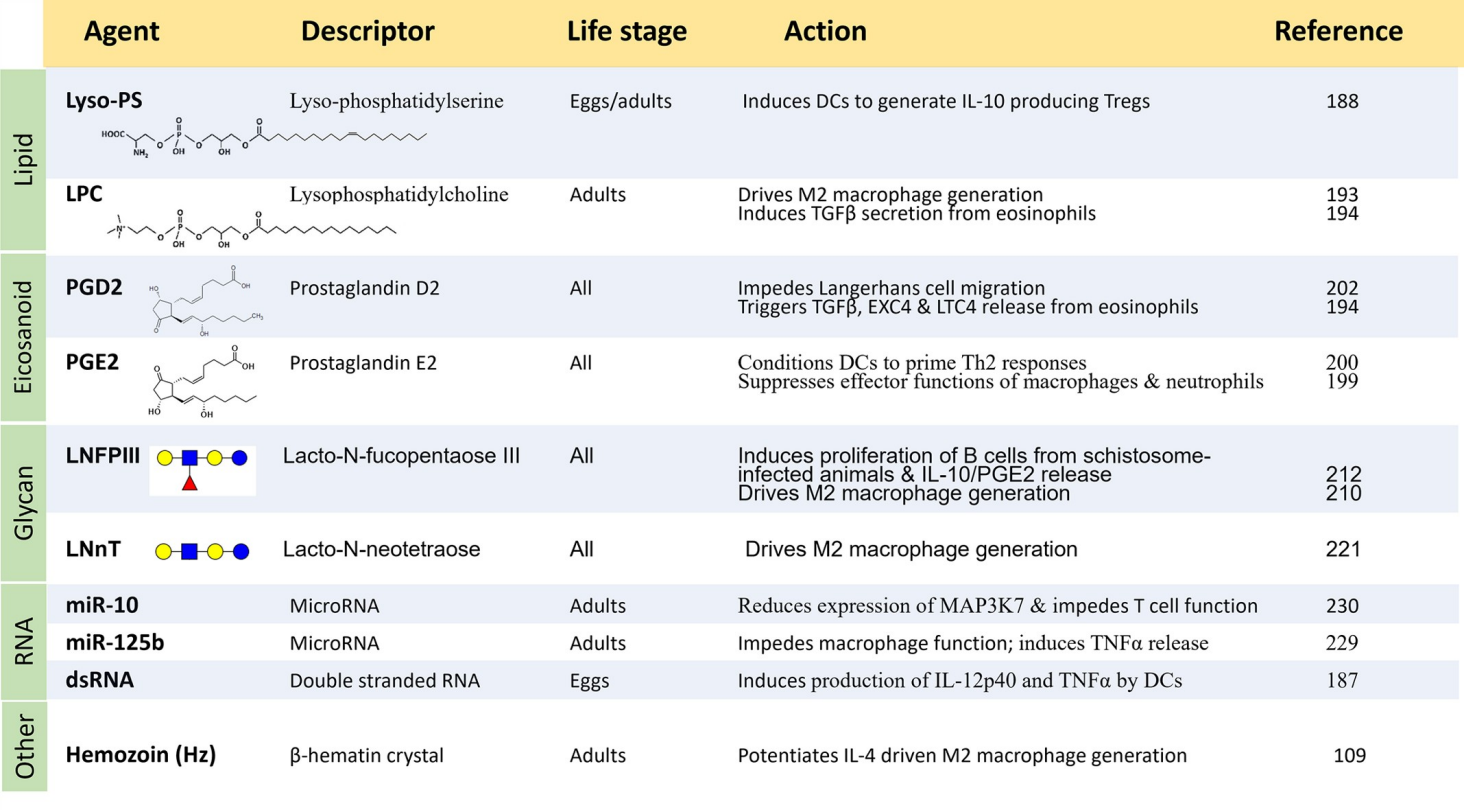
List of nonprotein immunomodulatory schistosome products and their major effects. Structural representations of some molecules are presented: For the glycans LNFPIII and LNnT, blue circles depict glucose, blue squares N-acetylglucosamine, yellow circles galactose, and the red triangle indicates fucose. Lyso-PS 20:1 and LPC 16:0 are depicted. DC, dendritic cell; EXC4, eoxin C4; LTC4, leukotriene C4; TGF-β, transforming growth factor beta; TNFα, tumor necrosis factor alpha; Treg, regulatory T cell.

In sum, it is clear that, throughout the life cycle of schistosomes, their mammalian hosts are exposed to a plethora of parasite biomolecules with which host cells interact. Some such molecules are exposed at the host parasite interface where they can impinge on host immunology in the local environment; other molecules are released from the worms as packages in the form of EVs and/or as a collection of excreted and/or secreted moieties (ES). Released molecules, traveling in the blood stream, can have local as well as more distal direct impact on host cells. Much work has examined the impact of individual parasite molecules on isolated cell types. However, Figs 1–3 make clear that multiple factors—some stimulatory, some inhibitory—could impinge on these cells at once. How these various inputs are integrated by host cells to yield the outcome seen following natural infection remains to be fully uncovered. Clearly, schistosomes employ a wide array of biomolecules—protein, lipid, glycan, nucleic acid, and more, in their efforts to bend host biochemistry to their liking. Fig 4 lists schistosome proteins that have been reported to have immunomodulatory impact, and Fig 5 lists nonprotein immunomodulators. Examining precisely how these, and other parasite molecules, modulate host metabolic and immunological pathways serves both to increase our understanding of host–pathogen interaction and may suggest new treatments against infection. Additionally, this knowledge is increasingly being used to develop interventions against immune, inflammatory, and other diseases—conditions completely unrelated to infection with a neglected tropical pathogen [28,42,43,117,156,167,233,234].
